# Combined Effect of Dipping in Oxalic or in Citric Acid and Low O_2_ Modified Atmosphere, to Preserve the Quality of Fresh-Cut Lettuce during Storage

**DOI:** 10.3390/foods9080988

**Published:** 2020-07-24

**Authors:** Bernardo Pace, Imperatrice Capotorto, Michela Palumbo, Sergio Pelosi, Maria Cefola

**Affiliations:** 1 Institute of Sciences of Food Production, National Research Council of Italy (CNR), c/o CS-DAT, Via Michele Protano, 71121 Foggia, Italy; imperatrice.capotorto@ispa.cnr.it (I.C.); michela.palumbo@ispa.cnr.it (M.P.); sergio.pelosi@ispa.cnr.it (S.P.); 2 Department of Science of Agriculture, Food and Environment, University of Foggia, Via Napoli 25, 71122 Foggia, Italy

**Keywords:** *Lactuca sativa* L., minimally processed lettuce, modified atmosphere packaging, oxalic acid, shelf life

## Abstract

Leaf edge browning is the main factor affecting fresh-cut lettuce marketability. Dipping in organic acids as well as the low O_2_ modified atmosphere packaging (MAP), can be used as anti-browning technologies. In the present research paper, the proper oxalic acid (OA) concentration, able to reduce respiration rate of fresh-cut iceberg lettuce, and the suitable packaging materials aimed to maintaining a low O_2_ during storage, were selected. Moreover, the combined effect of dipping (in OA or in citric acid) and packaging in low O_2_ was investigated during the storage of fresh-cut iceberg lettuce for 14 days. Results showed a significant effect of 5 mM OA on respiration rate delay. In addition, polypropylene/polyamide (PP/PA) was select as the most suitable packaging material to be used in low O_2_ MAP. Combining OA dipping with low O_2_ MAP using PP/PA as material, resulted able to reduce leaf edge browning, respiration rate, weight loss and electrolyte leakage, preserving the visual quality of fresh-cut lettuce until 8 days at 8 °C.

## 1. Introduction

Iceberg lettuce (*Lactuca sativa* L.) is considered one of the most popular vegetable and represent the primary fresh-cut product processed in Italy as well as in many other European and North America countries. Iceberg lettuce is highly desired by worldwide consumers and processors for its sensory and technological properties [1]. When lettuce is processed as fresh-cut product, an accelerated metabolism, due to the processing operations, causes, among the other alterations, the browning of cut surface [1,2,3,4]. Peroxidase (POD; EC 1.11.1.7) and polyphenol oxidase (PPO; EC 1.14.18.1) are enzymes that catalyze the conversion of polyphenols into quinones. In particular PPO catalyzes both hydroxylation of monophenols to o-diphenols and oxidation of colorless o-diphenols to o-quinones give brown pigments to cut tissues. While POD promote the oxidation of phenols to quinones in the presence of hydrogen peroxide [1]. To limit these changes that affect quality, supplementary techniques have been used in addition to the low temperature, such as modified atmosphere, chemical dipping based on different organic acids, edible coatings, heat treatments [1,5,6,7]. Saltveit [8] reports that modified atmosphere packaging (MAP) with low O_2_ and high CO_2_ was successfully applied to control browning specifically for the iceberg lettuce, whereas Gorny [9] shows that atmospheres with O_2_ values between 0.5 kPa and 3 kPa are recommended for iceberg lettuce. In addition, natural dipping in solutions based on ascorbic, citric, salicylic or oxalic acid, to prevent leaf edge browning can be used. These substances are recognized as safe for food applications and consumption, and are widely used for fresh-cut products [7,10,11]. Citric acid has been extensively used for its anti-browning activity in minimally processed fruits and vegetables [12] and specifically on fresh lettuce [5]. Moreover, it was also reported the efficiency of oxalic acid in preventing lettuce browning and extending the shelf life of fresh-cut fruits and vegetables [5,10,11,13,14,15,16].

Starting from these considerations, the goal of the present work is to study the combined effect of dipping in oxalic or citric acid and low O_2_ MAP to control the leaf edge browning of fresh-cut lettuce. To this aim, the proper oxalic acid concentration, and the suitable packaging material able to maintain an adequate low O_2_ concentration inside MAP bags were selected in specifically preliminary trials. Changes in sensory characteristics, respiration rate and chemical parameters (such as ammonium content, electrolyte leakage, phenolic content and *o*-quinones) during cold storage were investigated. To our knowledge, this is the first manuscript describing the effect of oxalic acid on the quality of fresh-cut iceberg lettuce.

## 2. Materials and Methods 

### 2.1. Plant Material and Sample Preparation

Iceberg lettuce heads (*Lactuca sativa* L.) were obtained from a local farm located in Foggia, at the same growing stage and were transported to the laboratory under refrigerated conditions in polystyrene boxes. Samples were kept in darkness for 24 h at 4 °C and at 85% relative humidity before being processed.

Lettuce heads were processed by removing outer leaves and the stem with stainless steel knives, then they were cut using a vegetable cutter (CL52 Robot Coupe, Vincennes-Cedex, France). Thus, the fresh-cut iceberg lettuce pieces obtained (3 × 4 cm) were pooled and blended, to minimize product heterogeneity, and were used for the experiments described below.

Two preliminary trials, the first aimed to select the proper oxalic acid (OA) concentration and the other finalized to the choice of the suitable packaging material to be used in the MAP, were carried out.

Then, based on results of preliminary trials, an experiment was conducted in order to show the combined effect of dipping in OA or citric acid and low O_2_ MAP on fresh-cut iceberg lettuce. 

#### 2.1.1. Preliminarily Trials

##### Selection of the Proper Oxalic Acid Concentration

Four lots of fresh-cut iceberg lettuce, obtained as reported above, were used. Each lot was washed, firstly, in tap water for 5 min at 5 °C, and subsequently dipped for 1 min at 5 °C in one of the three different OA concentrations: 1, 3, or 5 mM OA. Samples dipped in tap water were used as control (CTRL). After dipping, lettuce pieces belonging to each dipping treatment were immediately placed in a manual centrifuge and dried for about 1 min to remove the water excess. Then, lettuce pieces were placed in open polypropylene (PP) bags. A total of 36 bags (3 replicate × 4 treatments, 1 mM OA, 3 mM OA, 5 mM OA, or CTRL, × 3 storage time, 2, 4, and 7 days) were prepared. All bags, with about 150 g of fresh-cut lettuce each, were stored at 8 (±1) °C. At 0 days and at each storage time, all samples were analyzed for the respiration rate.

##### Selection of the Proper Packaging Material

In the second preliminary trial, three packaging materials were compared: polypropylene (PP, 30 µm thickness, OTR 1100 cm^3^·m^−2^ 24 h^−1^·bar^−1^), polypropylene/polyamide (PP/PA, 67 μm thickness, OTR 100 cm^3^·m^−2^ 24 h^−1^·bar^−1^), and PP/PA micro-perforated (PP/PA MP, 67 μm thickness) (Carton Pack Srl, Rutigliano, Italy).

Fresh-cut lettuce was dipped for 1 min at 5 °C in tap water and then centrifuged for about 1 min to remove water excess. In each bag (21 × 18 cm), 150 g of fresh-cut iceberg lettuce pieces were placed. For each packaging material (PP, PP/PA or PP/PA MP) 12 bags were prepared and sealed using a packaging machine (Boxer 50 GAS-Lavezzini, Fiorenzuola d’Arda, Italy) with an initial atmosphere of 3 kPa O_2_ and 97 kPa N_2_.

In addition, 12 open PP bags with 150 g of fresh-cut iceberg lettuce each were used as control (CTRL). All bags were stored at 8 °C for 6 days. Initially, and at each storage time (1, 2, 3 and 6 days), the visual quality of lettuce pieces was evaluated and the atmosphere composition inside packages was analysed.

#### 2.1.2. Combined Effect of Dipping in Oxalic or in Citric Acid and Low O_2_ MAP

The results of the preliminary trials were applied in the present experiment. Fresh-cut lettuce was washed in tap water for 5 min at 5 °C, and then dipped for 1 min at 5 °C in one of the following different solutions: 5 mM OA (the best concentration resulted from preliminary trial), or 1% citric acid (w:v) (CIT). Samples dipped in tap water in the same conditions, were used as control (CTRL). After dipping, lettuce pieces, belonging to each treatment (OA, CIT or CTRL) were immediately placed in a manual centrifuge for about 1 min to remove water excess. Dipped samples (OA, CIT or CTRL) were subsequently placed in PP/PA bags (21 × 18 cm) (the proper material resulted from preliminary trial) and sealed, with an initial modified atmosphere (MAP) of 3 kPa O_2_ and 97 kPa N_2_, using the packaging machine.

A total of 27 bags (3 replicates × 3 dipping treatments, OA, CIT, CTRL x 3 storage time, after 3, 6 and 8 days) were prepared. All bags, with about 150 g fresh-cut iceberg lettuce each, were stored at 8 (±1) °C. The quality parameters measured, initially, and at each storage time, are described below.

### 2.2. Respiration Rate and Headspace Analysis

The respiration rate of fresh-cut iceberg lettuce was measured at 8 °C using a closed system as previously descripted by Kader [17] initially and during storage (just after the opening of the bags for MAP samples). About 100 g of product, for each treatment and replicate (*n* = 3), was placed into 6 L sealed plastic jars (one jar per replicate) where CO_2_ was allowed to accumulate up to 0.1 kPa (concentration of the CO_2_ standard). The CO_2_ analysis was conducted taking 1 mL of gas sample from the headspace of the plastic jars and injecting into a gas chromatograph (p200 micro GC-Agilent, Santa Clara, CA, USA) equipped with dual columns and a thermal conductivity detector. Carbon dioxide was analyzed with a retention time of 16 s and a total run time of 120 s on a 10 m porous polymer (PPU) column (Agilent, Santa Clara, CA, USA) at a constant temperature of 70 °C. Respiration rate was expressed as mL CO_2_ kg^−1^·h^−1^.

Gas composition (O_2_ and CO_2_ kPa) of packages in MAP was measured during storage, using a gas analyser (CheckPoint O_2_/CO_2_ Dansensor^®^ Mocon, Ringsted, Denmark). 

### 2.3. Sensory Evaluation, Colour, Texture and Weight Loss

Fresh-cut iceberg lettuce belonging to each treatment was examined by a group of eight trained researchers at the beginning of the experiment (on the fresh lettuce) and at each storage time. Coded (3 digits) samples were presented to the trained researchers (judges) individually, to enable them to make independent evaluations.

Visual quality was evaluated on a 5-point rating scale according to Cefola et al. [18] where 5: excellent (fresh appearance, full sensory acceptability); 4: good (product acceptable from a sensory point of view); 3: limit of sensory acceptability; 2: product has notable visual defects; 1: severe visual defects. Samples rated below 3 were considered as unmarketable for the loss of the sensory visual quality (loss of turgor and brightness accompanied by softening and browning of leaf tissues). A color photographic scale, accompanying with a brief description of freshness, color uniformity, and brightness, was applied as reference.

The color of lettuce pieces was measured using a colorimeter (CR-400-Konica Minolta, Osaka, Japan) equipped with a D65 illuminant in the reflectance mode and in the CIE L* a* b* color scale. In detail, L* indicates the lightness from black (0 value) to white (100 value), a* the redness (+) or greenness (-), and b* the yellowness (+) or blueness (-).

The color was measured on three random points on the midribs surface of 5 pieces of lettuce for each replicate. The instrument was calibrated with a white plate as standard reference (L* = 97.55, a* = 1.32, b* = 1.41). The a* and b* color parameters recorded, were used for the calculation of the hue angle (h°) using the following formula [19].

(1) $$h^{{}^{\circ}}=arctg\frac{b*}{a*},$$

where, a* and b* are the color parameters acquired by colorimeter. 

The texture of the fresh-cut lettuce was assessed using a texture analyzer (ZwickLine Z0.5-Zwick/Roell, Ulm, Germany) equipped with a Kramer shear cell with 10 blades. For each replicate 5 samples of about 3 g each one were used. Texture was expressed as the maximum peak force (N) per gram of lettuce (N g^−1^).

The weight loss of each replicate was calculated as percentage compared with the initial weight.

### 2.4. Electrolyte Leakage and Ammonium Content

Electrolyte leakage was measured applying the procedure described by Kim et al. [20] with slight modifications. For each replicate, about 2.5 g of lettuce disks of 8 mm of diameter, obtained using a cork borer, were immersed in tubes containing 25 mL of distilled water. After 30 min of storage at 8 °C, the conductivity of the solution was measured by a conductivity meter (Cond. 51+, XS Instruments, Carpi, Italy). The tubes, with the vegetable portion, were then frozen and after 24 h, samples were thawed, and the total conductivity was measured. Electrolyte leakage was calculated as the percentage ratio of initial over total conductivity.

Ammonium content was evaluated according to Fadda et al. [21]. Lettuce pieces (5 g) were chopped and homogenized (T 25 digital Ultra-Turrax - IKA, Staufen, Germany) for 2 min in 20 mL of distilled water on an ice bath, and then centrifuged (Prism R C2500-R - Labnet, Edison, NJ, USA) for 5 min at 6440× *g* at 4 °C. Then, the supernatant extract (0.5 mL), was mixed with 5 mL of nitroprusside reagent (phenol and hypochlorite in alkali reaction mixture) and heated at 37 °C for 20 min. The color development after incubation, was determined with a spectrophotometer (UV-1800 - Shimadzu, Kyoto, Japan) reading the absorbance at 635 nm. The content of NH_4_^+^ was expressed as mmol NH_4_^+^ kg^−1^ of fresh weight, using ammonium sulfate as standard (0–10 μg mL^−1^, R^2^ = 0.99).

### 2.5. Total Phenol Content and O-Quinones Determination 

The total phenol content was determined according to the method of Fadda et al. [21]. Five grams of chopped lettuce for each replication was homogenized in a methanol:water solution (80:20) for 1 min and then centrifuged at 4 °C at 6440× *g* for 5 min. The absorbance was read after 2 h at 765 nm. The total phenol content was calculated based on the calibration curve of gallic acid and was expressed as milligrams of gallic acid per kg of fresh weight.

Soluble *o*-quinones of lettuce tissues, were extracted as described by Degl’Innocenti et al. [22] with low modifications. Five grams of tissues were homogenized with 20 mL methanol:water solution (80:20) for 1 min, filtered and centrifuged at 4 °C at 6440× *g* for 5 min. The supernatant was used directly to measure the soluble *o*-quinones at a wavelength of 437 nm. The result was expressed as the absorbance for 5 g of fresh weight.

### 2.6. Statistical Analysis 

For each preliminary trial, a two-way ANOVA for *p* ≤ 0.05 was performed to evaluate the effects of treatments, storage time and their interaction on quality parameters measured. As for the main experiment, a two-way ANOVA for *p* ≤ 0.05 was performed to evaluate the effects of combined treatments (dipping and packaging), storage time and their interaction on quality parameters. When the interaction between factors was significant, data were shown as graphs with mean values ± standard deviation. The statistical analysis was performed using the software STATISTICA 6.0 (StatSoft, Hamburg, Germany). 

## 3. Results

### 3.1. Oxalic Acid Concentration and Packaging Selection

**Figure 1:**
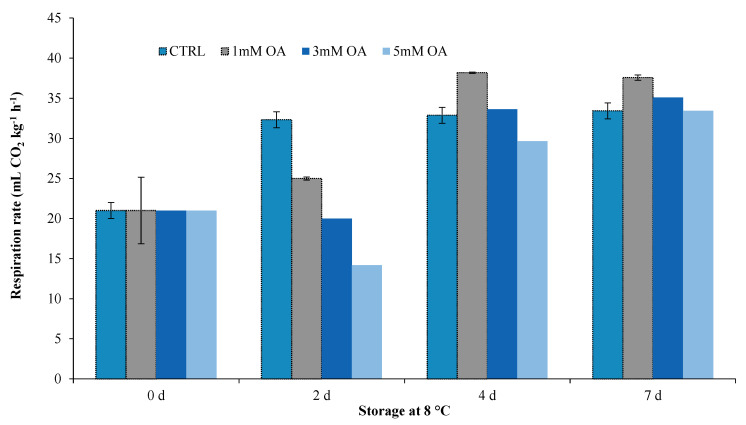
Respiration rate measured during the storage at 8 °C in fresh-cut iceberg lettuce dipped with different oxalic acid (OA) concentrations respect to control samples (CTRL).

In the first preliminary trial, three different OA concentrations were compared (1 mM, 3 mM or 5 mM) as fresh-cut lettuce dipping treatments for 1 min at 5 °C and the respiration rate was monitored during storage in air at 8 °C. Respiration rate was significantly affected by interaction between dipping treatments and storage time (*p* ≤ 0.001). Fresh-cut lettuces dipped in OA showed the lowest respiration rate during the entire storage (Figure 1). In particular, after 2 days of storage in air at 8 °C, fresh-cut lettuce dipped in 5 mM OA showed a respiration rate about 50% lower than CTRL. Then, after 4 and 7 days, no significant differences compared to CTRL were measured. A similar trend was also detected for the other two dipping solutions (1 or 3 mM), but in these cases the reduction in respiration rate respect to CTRL after 2 days of storage was about 22% and 38% for 1 and 3 mM OA, respectively.

**Figure 2:**
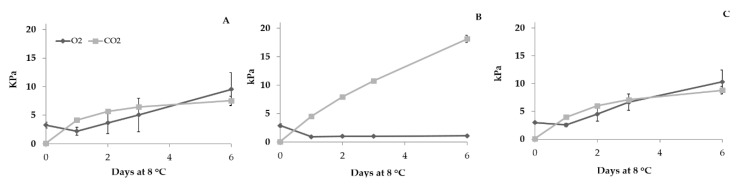
Changes in O_2_ and CO_2_ inside PP (**A**), PP/PA (**B**), PP/PA MP (**C**) fresh-cut iceberg lettuce bags. Data are mean (*n* = 3) ± standard deviation.

**Figure 3:**
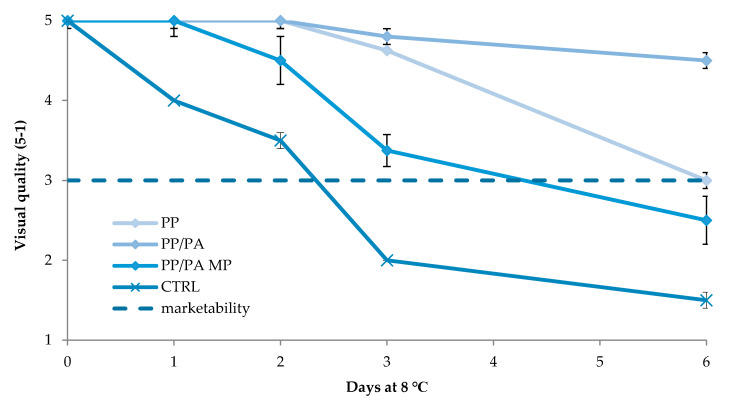
Changes in visual quality of fresh-cut iceberg lettuce stored in MAP using PP, PP/PA or PP/PA MP bags or in air (CTRL). Data are mean (*n* = 3) ± standard deviation.

Concerning the second preliminary trial, Figure 2 shows the changes in O_2_ and CO_2_ inside the bags closed in MAP with 3 kPa O_2_ and 97 kPa N_2_. In PP and PP/PA MP bags the O_2_ and CO_2_ increased during storage until to values between 5 and 10 kPa for both gases. On the other hand, in PP/PA bags the O_2_ concentration decreased until 1 kPa after 1 day of storage, then this value remained constant until the end of the trial, while the CO_2_ concentration increased reaching, at the end of the storage, a value of over 15 kPa. The gas composition measured inside PP/PA bags, positively affected the fresh-cut lettuce visual quality (Figure 3). Indeed, fresh-cut lettuce stored in PP/PA showed the highest visual quality score during the entire storage, followed by samples stored in PP, PP/PA MP and CTRL. Moreover, lettuce stored in air lost marketability after only 2 days of storage, mainly for the severe browning of the cut-surface. On the other hand, samples stored in MAP resulted marketable for 4–6 days in PP or PP/PA MP bags, while a long marketability (more than 6 days) might be reached when fresh-cut lettuce was packed in PP/PA. To sum up, the results of these two preliminary trials were used in the main experiment aimed to select the proper combination of dipping and MAP to improve fresh-cut lettuce marketability.

### 3.2. Combined Effect of Dipping and MAP to Extend the Marketability of Fresh-Cut Lettuce

Results of the two-way ANOVA showed a significant effect of interaction (combined treatment × storage) on visual quality, hue angle and respiration rate (Table 1). Total phenol and *o*-quinones were affected by storage and combined treatment, respectively, while weight loss, electrolyte leakage and ammonium were affected by both factors (Table 1). The main effect of each factor on these parameters (weight loss, electrolyte leakage, ammonium content, total phenols and *o*-quinones) are reported in Table 2. The product treated with OA-MAP showed, a significant lower mean value for weight loss, electrolyte leakage and o-quinones than the other combined treatments (Table 2).

The highest value of ammonium content was measured for CIT-MAP, whereas no differences on total phenol content was reported among treatments (Table 2).

Nonetheless, during storage, a significant increase in weight loss, ammonium content, and total phenols was measured in all samples, while for *o*-quinones no significant difference was observed (Table 2).

**Figure 4:**
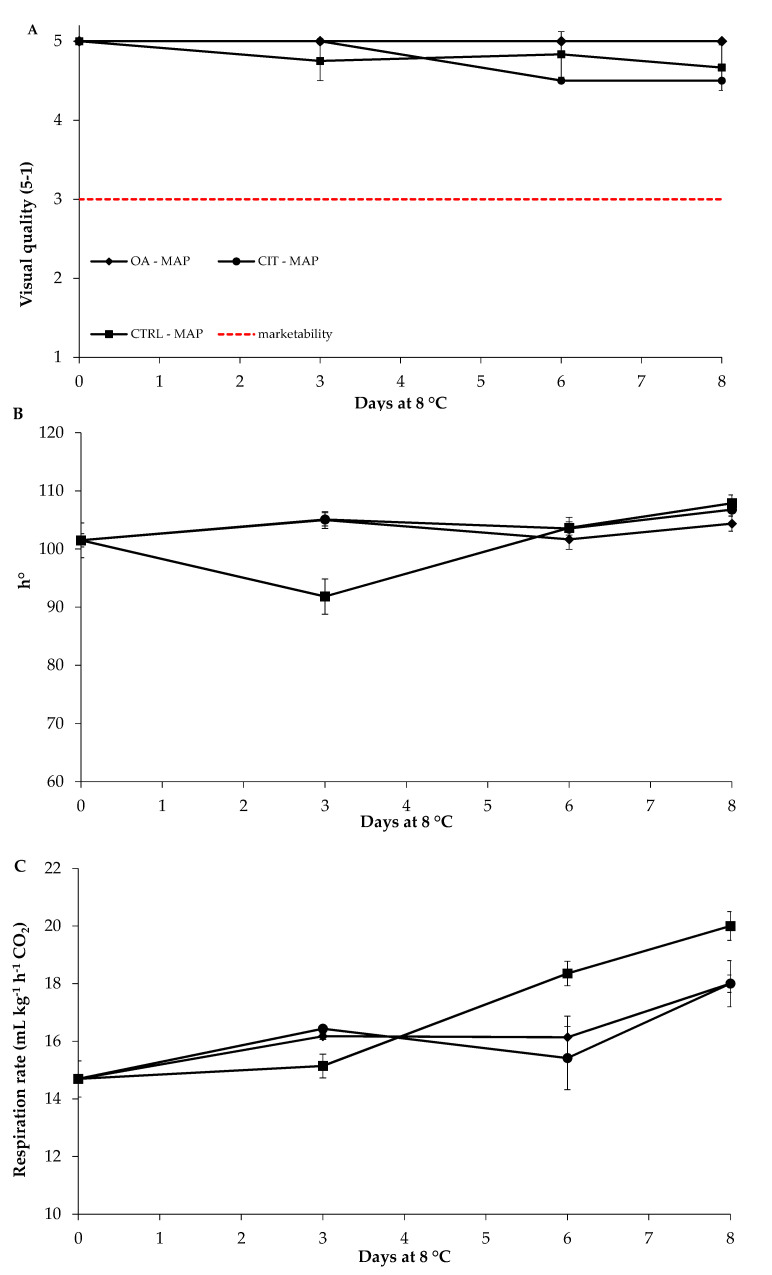
Changes in visual quality (**A**), hue angle (**B**), and respiration rate (**C**) in fresh-cut iceberg lettuce dipped in oxalic acid (OA), citric acid (CIT) or control (CTRL) and stored in modified atmosphere packaging (MAP). Data are mean value (*n* = 3) ± standard deviation.

In Figure 4, changes in VQ, hue angle and respiration rate during storage are reported. In particular, the VQ value, remained almost constant in sample OA-MAP, while a slight reduction was observed for CIT-MAP and CTRL-MAP after 3 and 6 days, respectively, although the mean values remained above the marketable limit (VQ = 3) for the entire trial in all samples (Figure 4A). As for h°, the initial value remained almost constant during storage for all samples and only in CTRL-MAP sample a slight reduction after 3 days of storage was measured (Figure 4B).

Starting from an initial value of about 15 mL CO_2_ kg^−1^ h^−1^, respiration rate increased in all samples over time: in details, OA-MAP and CIT-MAP showed a similar trend, with an increase at the end of the trial of about 30% compared to fresh samples, while the rise in CTRL-MAP was of roughly 45% (Figure 4C).

## 4. Discussion

Fresh-cut lettuce marketability is mainly affected by the leaf edge browning, due to the action of polyphenol oxidase (PPO) on phenols [23,24]. As a consequence, the shelf life of fresh-cut lettuce is limited to 2–3 days at 8 °C in air. In this research paper, the combined effect of dipping in oxalic acid or citric acid and MAP (3 kPa O_2_ in 97 kPa N_2_) was studied with the goal to extend the fresh-cut lettuce marketability.

The low PP/PA permeability to O_2_ and CO_2_ at 8 °C, promoted inside packages a flow of O_2_ adequate for product respiration. Thus, after a slight reduction from the initial value, the O_2_ concentration remained stable at 1 kPa, while CO_2_ increased to about 15 kPa. It was reported that O_2_ concentrations below 3 kPa, but not less than 0.5–1 kPa, avoid the fermentation and browning of the cut surface in fresh-cut lettuce [25]. These atmosphere conditions positively affected the storability of fresh-cut lettuce as previously observed [26]. Moreover, these concentrations are suitable to delay browning in fresh-cut lettuce due to the reduction in the phenolic metabolism [27,28,29].

The combined treatment based on dipping in OA and low O_2_ MAP allowed controlling respiration rate and weight loss, limiting the browning development and electrolyte leakage, finally preserving the visual quality. OA is generally recognized as safe (GRAS) compound, useful in pre and postharvest treatments on fruits and vegetables [15]. The positive effect of OA concentration applied on respiration rate and weight loss, might be due to a delay of metabolic activity, as reported for artichoke [30], asparagus [13], and rocket and baby spinach leaves [11]. In addition, Cefola et al. [11] reported that OA dipping might act as antioxidant controlling vegetable tissue browning as well as preserving the leaf membrane integrity, limiting the increase in electrolytic leakage during storage [16]. It was reported that OA might directly inhibit the activities of the enzymes involved in browning reactions (PPO, POD), reducing the pH of the treated produce. On the other hand, OA could react with soluble quinones, reducing them to into uncoloured catechol or creating colourless adducts [6,31].

## 5. Conclusions

Oxalic acid 5 mM was selected as proper concentration able to delay the respiration rate of fresh-cut iceberg lettuce. Moreover, suitable packaging was obtained using polypropylene/polyamide bags closed in modified atmosphere packaging with 3 kPa O_2_ in 97 kPa N_2_. Combining dipping in oxalic acid or citric acid with low O_2_ modified atmosphere packaging, the best results were obtained using oxalic acid as treatment. In particular, the combined effect of oxalic acid dipping and low O_2_ modified atmosphere packaging allowed us to reduce the respiration rate, weight loss, and electrolyte leakage during storage, preserving the visual quality of fresh-cut iceberg lettuce stored at 8 °C until 8 days.

## Figures and Tables

**Table 1:** Effect of combined treatments (dipping in oxalic acid, citric acid or water and packaging in MAP) (A), storage (3, 6, 8 days at 8 °C) (B), and their interaction (A × B) on quality parameters of fresh-cut iceberg lettuce.

Parameter	Combined Treatment (A)	Storage (B)	Combined Treatment × Storage (A × B)
Respiration rate (mL CO_2_ kg^−1^·h^−1^)	ns	*	***
Hue angle (h°)	***	***	***
Texture (N·g^−1^)	ns	ns	ns
Weight loss %	*	***	ns
Visual Quality (VQ)	***	ns	*
Off Odour	ns	ns	ns
Electrolyte leakage %	*	*	ns
Ammonium content (mmol·kg^−1^ NH_4_^+^)	**	***	ns
Total phenols (mg·kg^−1^ gallic acid)	ns	*	ns
*o*-quinones (OD 427 nm)	*	ns	ns

Asterisks indicate the significance level for each factor of the ANOVA test (ns, not significant; * *p* ≤ 0.05; ** *p* ≤ 0.01; *** *p* ≤ 0.0001).

**Table 2:** Main effects of main factors (combined treatments or storage) on the postharvest quality of fresh-cut iceberg lettuce.

Main Factors	Weight Loss %		Electrolyte Leakage %		Ammonium Content (mmol·kg^−1^ NH_4_^+^)		Total Phenols (mg·kg^−1^ gallic acid)		*O*-Quinones (OD 427 nm)	
**Combined Treatments**										
OA-MAP	0.361	b	41.4	b	1.99	b	83.1	ns	0.10	b
CIT-MAP	0.445	ab	44.3	a	2.17	a	89.6	ns	0.12	ab
CTRL-MAP	0.512	a	44.1	a	1.96	b	83.9	ns	0.14	a
Storage										
3	0.295	b	42.5	b	1.72	b	76.4	b	0.13	ns
6	0.484	a	45.2	a	2.15	a	88.6	a	0.11	ns
8	0.539	a	42.1	b	2.27	a	91.7	a	0.13	ns

For each factor (combined treatments or storage) different letters (a,b) indicate significant differences (*p* < 0.05) according to Dunkan’s test. ns, not significant.

## References

[1] Pace B., Capotorto I., Ventura M., Cefola M. (2015). Evaluation of L-cysteine as anti-browning agent in fresh-cut lettuce processing. J. Food Process. Preserv..

[2] Heimdal H., Kühn B.F., Poll L., Larsen L.M. (1995). Biochemical changes and sensory quality of shredded and MA-packaged iceberg lettuce. J. Food Sci..

[3] Amodio M.L., Cefola M., Pace B., Colelli G., Tonetto de Freitas S., Pareek S. (2019). Fresh-Cut Fruits and Vegetables. Postharvest Physiological Disorders in Fruits and Vegetables.

[4] Tudela J.A., Gil M.I., Gil M.I., Beaudry R. (2020). Leafy vegetables: Fresh-cut lettuce. Controlled and Modified Atmospheres for Fresh and Fresh-Cut Produce.

[5] Altunkaya A., Gökmen V. (2008). Effect of various inhibitors on enzymatic browning, antioxidant activity and total phenol content of fresh lettuce (*Lactuca sativa*). Food Chem..

[6] Altunkaya A., Gökmen V. (2009). Effect of various anti-browning agents on phenolic compounds profile of fresh lettuce (*L. sativa*). Food Chem..

[7] Soares C.D.F., Martin J.G.P., Berno N.D., Kluge R.A. (2019). Antioxidant chemical treatment affects physiology and quality of minimally-processed escarole. Horticulturae.

[8] Saltveit M.E., Tomás-Barberán F.A. (1997). Physical and physiological changes in minimally processed fruits and vegetables. Phytochemistry of Fruit and Vegetables.

[9] Gorny J.R. (2003). A summary of CA and MA requirements and recommendations for fresh-cut (minimally processed) fruits and vegetables. Acta Hort..

[10] Suttirak W., Manurakchinakorn S. (2010). Potential application of ascorbic acid, citric acid and oxalic acid for browning inhibition in fresh-cut fruits and vegetables. Walailak J. Sci. Tech..

[11] Cefola M., Pace B. (2015). Application of oxalic acid to preserve the overall quality of rocket and baby spinach leaves during storage. J. Food Process. Preserv..

[12] Ahvenainen R. (1996). New approaches in improving the shelf life of minimally processed fruit and vegetables. Trends Food Sci. Technol..

[13] Barberis A., Cefola M., Pace B., Azara E., Spissu Y., Serra P.R., Logrieco A.F., D’hallewin G., Fadda A. (2019). Postharvest application of oxalic acid to preserve overall appearance and nutritional quality of fresh-cut green and purple asparagus during cold storage: A combined electrochemical and mass-spectrometry analysis approach. Postharvest Biol. Technol..

[14] Zheng J., Li S., Xu Y., Zheng X. (2019). Effect of oxalic acid on edible quality of bamboo shoots (Phyllostachys prominens) without sheaths during cold storage. LWT.

[15] Ali S., Khan A.S., Anjum M.A., Nawaz A., Naz S., Ejaz S., Hussain S. (2020). Effect of postharvest oxalic acid application on enzymatic browning and quality of lotus (Nelumbo nucifera Gaertn.) root slices. Food Chem..

[16] Zheng X.L., Brecht J.K., Pareek S. (2018). Oxalic acid treatments. Novel Postharvest Treatments of Fresh Produce.

[17] Kader A.A., Kader A.A. (2020). Methods of gas mixing, sampling and analysis. Postharvest Technology of Horticultural Crops.

[18] Cefola M., Carbone V., Minasi P., Pace B. (2016). Phenolic profiles and postharvest quality changes of fresh-cut radicchio (*Cichorium intybus* L.): Nutrient value in fresh vs. stored leaves. J. Food Comp. Anal..

[19] Cefola M., D’Antuono I., Pace B., Calabrese N., Carito A., Linsalata V., Cardinali A. (2012). Biochemical relationships and browning index for assessing the storage suitability of artichoke genotypes. Food Res. Int..

[20] Kim J.G., Luo Y., Tao Y., Saftner R.A., Gross K.C. (2005). Effect of initial oxygen concentration and film oxygen transmission rate on the quality of fresh-cut romaine lettuce. J. Sci. Food Agric..

[21] Fadda A., Pace B., Angioni A., Barberis A., Cefola M. (2016). Suitability for ready-to-eat processing and preservation of six green and red baby leaves cultivars and evaluation of their antioxidant value during storage and after the expiration date. J. Food Process. Pres..

[22] Degl’Innocenti E., Pardossi A., Tognoni F., Guidi L. (2007). Physiological basis of sensitivity to enzymatic browning in “lettuce”, “escarole” and “rocket salad” when stored as fresh-cut products. Food Chem..

[23] Zhou T., Harrison A.D., McKellar R., Young J.C., Odumeru J., Piyasena P., Lu X., Mercer D.G., Karr S. (2004). Determination of acceptability and shelf life of ready-to-use lettuce by digital image analysis. Food Res. Int..

[24] Pace B., Cefola M., Da Pelo P., Renna F., Attolico G. (2014). Nondestructive evaluation of quality and ammonia content in whole and fresh-cut lettuce by computer vision system. Food Res. Int..

[25] Martínez-Sánchez A., Tudela J.A., Luna C., Allende A., Gil M.I. (2011). Low oxygen levels and light exposure affect quality of fresh-cut Romaine lettuce. Postharvest Biol. Technol..

[26] Cantwell M., Suslow T. (2001). Lettuce: Crisphead or Iceberg. Recommendations for Maintaining Postharvest Quality. http://ucanr.edu/sites/Postharvest_Technology_Center_/Commodity_Resources/Fact_Sheets/Datastores/Vegetables_English/?uid=19&ds=799.

[27] Lopez-Gàlvez G., Salveit M., Cantwell M. (1996). The visual quality of minimally processed lettuces stored in air or controlled atmosphere with emphasis on romaine and iceberg types. Postharvest Biol. Technol..

[28] Gorny J.R. A summary of CA and MA requirements and recommendations for fresh-cut (minimally processed) fruits and vegetables. Proceedings of the 7th International Controlled Atmosphere Conference, University of California.

[29] Hertog M.L.A.T., Peppelenbos H.W., Evelo R.G., Tijskens L.M.M. (1998). A dynamic and generic model of gas exchange of respiring produce: The effects of oxygen, carbon dioxide and temperature. Postharvest Biol. Technol..

[30] Ruíz-Jiménez J.M., Zapata P.J., Serrano M., Valero D., Martínez-Romero D., Castillo S., Guillén F. (2014). Effect of oxalic acid on quality attributes of artichokes stored at ambient temperature. Postharvest Biol. Technol..

[31] Ali H.M., El-Gizawy A.M., El-Bassiouny R.E., Saleh M.A. (2016). The role of various amino acids in enzymatic browning process in potato tubers, and identifying the browning products. Food Chem..

